# Renal injury due to vitamin D intoxication; a case of dispensing error

**DOI:** 10.12861/jrip.2013.27

**Published:** 2013-06-01

**Authors:** Hamid Nasri, Muhammed Mubarak

**Affiliations:** ^1^Department of Nephrology, Division of Nephropathology, Isfahan University of Medical Sciences, Isfahan, Iran; ^2^Department of Histopathology, Sindh Institute of Urology and Transplantation (SIUT), Karachi, Pakistan

**Keywords:** Acute kidney injury, Hypercalcemia, Hypervitaminosis D, Medication error, Vitamin D toxicity, Chronic Kidney Disease

**Implication for health policy/practice/research/medical education:**

Vitamin D has become a popular drug nowadays and is commonly prescribed for a variety of illnesses. Dosage, prescription and dispensing errors are common and can lead to vitamin D toxicity. The diagnosis of the later is often delayed and can lead to irreversible damage to a number of organs including kidneys. It is important to educate both the health care providers and the patients on the benefits and risks of use of vitamin D and inform them of the safety measures to avoid overdosing of the drug. Vitamin D intoxication can lead to acute or chronic renal injury and this cause should be considered in differential diagnosis of unusual cases of renal failure and hypercalcemia.

## Case presentation

A 56-year-old man, Afghan origin, with a history of significant weight reduction in a period of three months (about 20 kg), was admitted to the hospital. In the past medical history, one year before hospitalization, he had the history of low back pain and underwent disk hernia operation in India. Six months after disc hernioraphy, low back pain continued and the patient came back to India for further evaluation. On the second visit and evaluation, the patient’s laboratory tests revealed normal complete blood count (CBC), normal liver and renal function tests. Erythrocyte sedimentation rate (ESR) was 15 mm/1^st^ hr. Also the serum calcium, phosphorus, parathyroid hormone (PTH), serum protein, electrophoresis and upper gastrointestinal endoscopy were normal. At this time, the patient was prescribed six Vitamin D ampoules (300,000 IU), however, the drugstore gave him 60 Vitamin D ampoules. After returning to the country of origin (Afghanistan), he injected 40 Vitamin D ampoules (one vitamin D ampoule each week). During this period of time, the patient had 30 Kg weight loss and then returned to Iran and was admitted in our hospital. Besides weight loss, he also had the complaint of pruritus, nausea and vomiting. Primary evaluation revealed a serum creatinine of 4 mg/dl, serum calcium of 12 mg/dl, serum phosphorus of 3 mg/dl, serum intact PTH of 2.7 ng/ml (normal: 10-65 ng/ml), and vitamin D level of >400 nmol/l (normal: 47.7-144 nmol/l). Also the patient had serum hemoglobin of 9.8 g/dl, hematocrit of 30% and ESR of 55 mm/1^st^ hr. For further evaluation, a bone marrow aspiration and biopsy were performed, which were normal. Moreover, the results of serum protein electrophoresis, urine and serum immunoglobulin electrophoresis were normal. The serum angiotensin converting enzyme (ACE) and PSA level was normal. PSA as well as skull, chest and pelvic X-rays were normal. To find further information on the renal damage a renal biopsy was also performed. On light microscopy, the glomeruli had normal morphology and architecture (Figure 1A-D). There was no vasculopathy. The main pathology was found in the tubulointerstitial compartment. The interstitial area harbored significant inflammatory cell infiltration, predominantly mononuclear cells, associated with mild tubular atrophy and interstitial fibrosis. Both tubular dilatation and calcified necrotic debris in the tubular lumina were evident. Tubular cell degeneration was also seen (Figure 2A-D). On immunofluorescence study, there was no deposition of IgA, IgG, IgM, C3, C1q or fibrin. The diagnosis was tubulointerstitial nephritis, mostly chronic and compatible with the clinical diagnosis of vitamin D intoxication due to dispensing error. The patient was treated for hypercalcemia. Prednisolone, 1 mg/kg was started and gradually tapered. Serum creatinine and calcium level gradually decreased. However, serum vitamin D level continued to be high, even after one year. The patient returned to his country with serum creatinine of 1.5 mg/dl and calcium of 10 mg/dl. A low dose prednisolone (7.5 mg/day) was continued. We were updated on his laboratory results regularly. After two years, the last serum creatinine test showed a value of 1.2 mg/dl.

**Figure 1:**
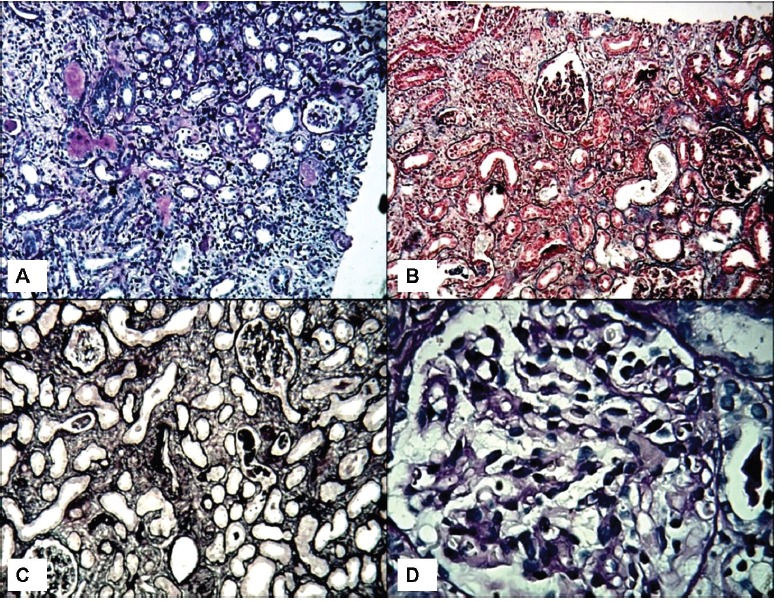
The morphological features seen on light microscopic study of the renal biopsy. A) Low-power view showing predominant pathology affecting the tubulointerstitial component. A portion of a glomerulus appears unremarkable. (Silver-periodic acid-Schiff (PAS), ×50). B) The glomeruli are essentially normal on light microscopy. The tubules show widespread degenerative changes with luminal dilatation. (Masson’s trichrome stain, ×100). C) Silver staining also highlighting minor changes in the glomeruli. There is however mild tubular atrophy and interstitial fibrosis. One arteriole in the center of the field is unremarkable. (Jones methenamine silver stain, ×100). D) High-power view showing in detail the essentially normal morphological appearances of a representative glomerulus from the biopsy. (Silver-PAS, ×200).

**Figure 1:**
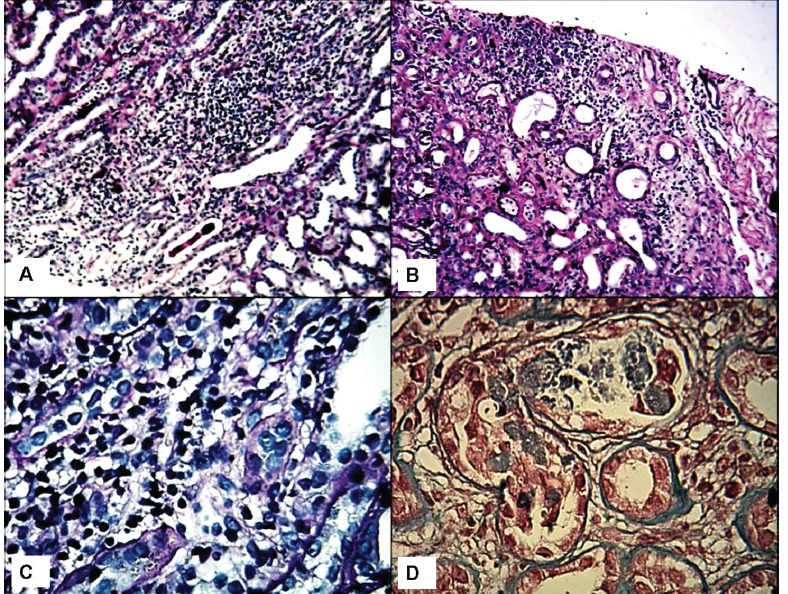
The detailed morphological features of tubulointerstitial injury of the kidney. A) Low-power view of a representative field showing prominent mononuclear inflammatory cell infiltration in the interstitium. (Silver-periodic acid-Schiff (PAS), ×100). B) Another area from the biopsy showing tubular atrophy, dilatation of tubular lumina and chronic inflammatory cell infiltration in the interstitium. (PAS, ×100). C) High-power view showing the nature of inflammatory cell infiltrates in the interstitium. These almost entirely consist of mononuclear cells. (Silver-PAS, ×200). D) High-power view of another representative area of the biopsy with degenerative changes of the tubular epithelial cells with luminal dilatation and dystrophic calcification. (Masson’s trichrome stain, ×400).

## Discussion

Vitamin D intoxication has been reported more frequently in recent years (1,2). Intoxication of vitamin D is a known cause of hypercalcemia and renal injury (1-3). The overzealous use of vitamin D in some countries due to the risk of rickets can lead to vitamin D intoxication. Vitamin D has a wide therapeutic index, its toxicity is well known and cases of accidental ingestion, malpractice, self-medication, and have been described. We report here a case of dispensing error-related vitamin D intoxication in an elderly male who presented with hypercalcemia, acute kidney injury, and significant weight loss (1-4). The symptoms and findings associated with vitamin D intoxication are closely linked to serum calcium level and duration of hypercalcemia (1-6). The diagnosis is often delayed because the presenting symptoms are often non-specific resulting from hypercalcemia — nausea, vomiting, weight loss, thirst, constipation, polyuria, headache, weakness and apathy (3-7). When suspected, the diagnosis is confirmed by the presence of hypercalcemia and evidence of kidney injury, the later may be acute or chronic. While vitamin D stimulates phosphate absorption too, serum phosphate is often normal, as in our case, unless the kidney impairment is severe. Reduction of serum calcium by administration of corticosteroids distinguishes this cause of hypercalcemia from hyperparathyroidism, while the action of corticosteroids is to inhibit the conversion of 25-(OH) vitamin D to 1, 25-(OH)2 vitamin D (3-9). In this case, the assessment of 25-(OH)-vitamin D level, which continued to be high for at least 1.5 years of evaluation, confirmed the diagnosis of interstitial nephritis due to overdose of Vitamin D and resultant hypercalcemia. In our patient, continuing the corticosteroid therapy might also have been responsible for decreasing the inflammatory infiltration of renal interstitium and improvement of renal function. However, the second renal biopsy for further evaluation was not possible (4-11).

In conclusion, this case underscores the need for proper education of health care providers and patients on the advantages and risks associated with vitamin D supplementation and be informed of safety measures to avoid the overdosing of the drug. In addition, the case also highlights the need for extreme caution when using unregulated injectable form of vitamin D.

## Authors’ contributions

MM and HN wrote the manuscript equally.

## Ethical considerations

Ethical issues (including plagiarism, misconduct, data fabrication, falsification, double publication or submission, redundancy) have been completely observed by the author.

**Conflict of interest**s

The author declared no competing interests.

## Funding/Support

None.

## References

[1] Ozkan B, Hatun S, Bereket A (2012). Vitamin D intoxication. Turk J Pediatr.

[2] Jacobus CH, Holick MF, Shao Q, Chen TC, Holm IA, Kolodny JM (1992). Hypervitaminosis associated with drinking milk. N Engl J Med.

[3] Ross AC, Manson JE, Abrams SA, Aloia JF, Brannon PM, Clinton SK (2011). The 2011 report on dietary reference intakes for calcium and vitamin D from the Institute of Medicine: what clinicians need to know. J Clin Endocrinol Metab.

[4] Jamieson MJ (1985). Hypercalcaemia. Br Med J (Clin Res Ed).

[5] Allgrove J (2003). Disorders of calcium metabolism. Curr Paediatr.

[6] Jeon US (2008). Kidney and calcium homeostasis. Electrolyte Blood Press.

[7] Lafferty FW (1991). Differential diagnosis of hypercalcemia. J Bone Miner Res.

[8] Araki T, Holick MF, Alfonso BD, Charlap E, Romero CM, Rizk D (2011). Vitamin D intoxication with severe hypercalcemia due to manufacturing and labeling errors of two dietary supplements made in the United States. J Clin Endocrinol Metab.

[9] Allen SH, Shah JH (1992). Calcinosis and metastatic calcification due to vitamin D intoxication. Horm Res.

[10] Misra M, Pacaud D, Petryk A, Collett-Solberg PF, Kappy M (2008). Drug and Therapeutics Committee of the Lawson Wilkins Pediatric Endocrine SocietyVitamin D deficiency in children and its management: review of current knowledge and recommendations. Pediatrics.

[11] Jones G (2008). Pharmacokinetics of vitamin D toxicity. Am J Clin Nutr.

